# Pickles

**Published:** 1878-08

**Authors:** 


PICKLES.—"An excellent way to make pickles which will keep a year or more is—drop them into boiling hot water, but not boil them ; let them stay ten minutes, wipe them dry, then drop them into cold spiced vinegar. They will not need to be put into salt and water."

